# Differential Diagnostic Reasoning Method for Benign Paroxysmal Positional Vertigo Based on Dynamic Uncertain Causality Graph

**DOI:** 10.1155/2020/1541989

**Published:** 2020-01-24

**Authors:** Chunling Dong, Yanjun Wang, Jing Zhou, Qin Zhang, Ningyu Wang

**Affiliations:** ^1^School of Computer Science and Cybersecurity, Communication University of China, Chaoyang District, Beijing 100024, China; ^2^Department of Computer Science and Technology, Tsinghua University, Haidian District, Beijing 100084, China; ^3^Department of Otorhinolaryngology Head and Neck Surgery, Beijing Chaoyang Hospital, Captical Medical University, Beijing 100020, China

## Abstract

The accurate differentiation of the subtypes of benign paroxysmal positional vertigo (BPPV) can significantly improve the efficacy of repositioning maneuver in its treatment and thus reduce unnecessary clinical tests and inappropriate medications. In this study, attempts have been made towards developing approaches of causality modeling and diagnostic reasoning about the uncertainties that can arise from medical information. A dynamic uncertain causality graph-based differential diagnosis model for BPPV including 354 variables and 885 causality arcs is constructed. New algorithms are also proposed for differential diagnosis through logical and probabilistic inference, with an emphasis on solving the problems of intricate and confounding disease factors, incomplete clinical observations, and insufficient sample data. This study further uses vertigo cases to test the performance of the proposed method in clinical practice. The results point to high accuracy, a satisfactory discriminatory ability for BPPV, and favorable robustness regarding incomplete medical information. The underlying pathological mechanisms and causality semantics are verified using compact graphical representation and reasoning process, which enhance the interpretability of the diagnosis conclusions.

## 1. Introduction

Benign paroxysmal positional vertigo (BPPV) is a major cause of vertigo and accounts for approximately 17–42% of the cases. It has a lifetime prevalence of 2.4% in the general population [1]. BPPV is caused by displaced otoconia in the semicircular canal, and its clinical characteristics include a brief, episodic, and position-provoked vertigo. BPPV typically causes balance disturbance which considerably increases the risk of falls in patients. Furthermore, it causes malignant secondary damage, especially for elderly people [2]. The commonly used therapy for BPPV is the particle repositioning maneuver (PRM), also known as the canalith repositioning procedure. Theoretically, almost all patients can be readily treated with accurate diagnosis and pathogenesis [3, 4]. However, the average resolution rate with one PRM treatment for posterior semicircular canal BPPV in many trials was revealed to be only 8.6%. Notably, there are various treatment options; therefore, the effectiveness of PRM (such as the Epley and Semont maneuvers) depends on the accurate classification of BPPV. The classification process would include determining the underlying pathogeny, locating the calcium carbonate debris and identifying whether the material is free-floating or adherent to the cupula, among others. BPPV can be classified into many subtypes in clinical practice, such as the idiopathic BPPV and secondary BPPV, or the typical BPPV and atypical BPPV. However, distinguishing among several causes of vertiginous disorders with similar symptoms is quite complex. Therefore, clinical subtype differentiation of BPPV is important for effective repositioning maneuvers in treatment to reduce inappropriate vestibular suppressant medications and minimize unnecessary ancillary tests.

In recent years, some automatic multiaxial positioning devices, electronystagmograms, and videonystagmography have been applied in the management of BPPV [5]. These applications have digitized and standardized, laying a foundation for computer-aided intelligent diagnosis and clinical decision-making. Medical data typically contain hidden and complicated patterns and causality semantics; thus, appropriate encoding and interpretation of medical data result in better diagnosis, medicine, and treatment [6, 7]. Numerous research has been done on this; for instance, in the management of vertigo, there is a wide spectrum of potential ailments and intricate confounding factors which complicates the accuracy of identifying the different types of vertiginous disorders. Based on this, several otoneurological expert systems including VERTIGO [8], Carnisel [9], and otoneurological expert system (ONE) [10, 11] were developed. Notably, ONE is a mature system that has been validated in practice, and its inference approach resembles methods of weighted k-nearest neighbor (k-NN), genetic algorithm (GA), fuzzy logic, and decision tree.

Previous attempts have been made at exploring methods for automatic differential diagnosis of diseases. A method for differentiating pancreatic serous from mucinous cystadenomas based on morphological features extraction was proposed in [12]; this approach utilized classifiers such as Bayesian, k-NN, support vector machine (SVM), and artificial neural network (ANN). Furthermore, a method for integrating Hopfield networks, retrieval networks, and k-NN to mine the medical data for differential diagnosis was proposed in [6]. Moreover, Lopes et al. developed a decision-support system based on the fuzzy cognitive map to discriminate the diagnoses of alterations in urinary elimination [13]. A supervised machine learning algorithm based on the combination of principal components analysis and SVM for the differential diagnosis of Parkinsonian syndrome (PS) was proposed in [14]. Mudali et al. [15] studied the classification of PS using decision trees based on the scaled subprofile model and principal component analysis methods. Ota et al. [16] proposed a differential diagnosis tool for PS by applying the discriminant techniques derived by stepwise methods. The SVM and logistic regression-based models were developed in [17] for the classification and prediction of PS. A method based on the Bayesian network (BN) was proposed in [18] to perform classification of early glaucoma and cluster data into different stages of the disease. Moreover, multidimensional BN classifiers are proposed to assist the treatment of multiple sclerosis [19]. A BN-based method was used for classification of EEG-based multiclass motor imagery BCI [20]. Furthermore, a method was proposed in [21] for individualized characterization and diagnosis of cognitive impairments. Melin et al. [22] described a method of competitive neural networks and the learning vector quantization algorithm for classification of electrocardiogram signals. Finally, an expert system was provided in [23] for the differential diagnosis of strabismus based on the architecture of ANN and the Levenberg–Marquardt back propagation method.

There are several challenges in this field of research. First, it is difficult to faithfully model the pathogenesis and pathophysiology characterized by individual differences, and uncertain factors including intricate and confounding disease factors, and insufficient sample data, among others. To address this problem, we believe that medical knowledge and experience could play an important role. The knowledge derived from clinical data and that specified by the domain experts should be integrated to provide a basis for practical decision-making [24, 25]. The introduction of expert knowledge has been further recognized as an effective solution for reducing the inherent uncertainty of the models based on automatic learning methods [26].

Second, many diagnosis approaches are mostly deficient in handling incomplete examinations and tests in clinical practice. The dependence of the models on the completeness of medical data limits some methods' functions in cases where the information is incomplete or imperfect. Some approaches have been proposed to deal with cases of missing data in the diagnosis of chronic obstructive pulmonary disease; these include evaluating the similarity of attributes with unknown values or filling the gaps statistically with plausible values [27]. Although there has been research on the plugging missing values by identifying the regularities in the occurrence model, very few studies have been devoted to effectively improving the diagnostic reasoning method in cases where there is incomplete information. Notably, in disease diagnosis, using incomplete information to accurately diagnose a disease reduces the need for unnecessary medical examinations.

Third, although the probability is known to be capable of providing a sound and flexible procedure for interpreting uncertain evidence with which the clinical practice is confronted, it is difficult to make a conclusion readily comprehensible to the users. It is important to determine the underlying etiology and pathology for disease occurrences, the process of systematic diagnosis, and a means of confirming that the right conclusion has been reached. Therefore, an explicable diagnostic reasoning model that matches the method for drawing conclusions is essential. Furthermore, undesirable interpretability or explicability is a common hinderance for the practical application of most methods.

In summary, it is well known that distinguishing the wide range of vertigo causes is a complicated endeavor, even for experienced physicians. Even though BPPV is suggested in the diagnostic reasoning outcome, the subtype cannot be easily discriminated. In our opinion, an in-depth understanding and appropriate representation of the pathogenesis and pathophysiology characterized by individual differences contribute to the diagnostic accuracy. This study, therefore, seeks to develop an efficient and easy-to-implement differential diagnosis method for BPPV based on the dynamic uncertain causality graph (DUCG). The main focus of the proposed method is solving the three aforementioned problems of the knowledge-based modeling, incompleteness of the medical data, and interpretability of the method.

The knowledge representation and inference methods of DUCG are introduced in [28–31]. In recent years, DUCG has been applied in several medical diagnostic systems involving multiple diseases such as jaundice, syncope, and sellar region disease, among others. Our earlier work in [30] presented a modeling and reasoning methodology for vertigo diagnosis and developed a decision-support system which covered 22 common vertigo diseases. However, the methods are not completely applicable to the differential diagnosis problems. Therefore, this paper proposes new algorithms for reasoning about BPPV subtype differentiation.

The structure of this article is as follows: Section 2 describes the proposed method based on DUCG, including the uncertain causality representation, the BPPV model, and the differential diagnostic reasoning algorithms. In Section 3, a series of verification experiments using clinical cases are presented, followed by a comparative analysis and an application analysis. Finally, Section 4 contains the conclusions.

## 2. Methods

### 2.1. DUCG Causality Representation and Diagnostic Reasoning Method

#### 2.1.1. Uncertain Causality Representation Scheme

**Figure 1:**
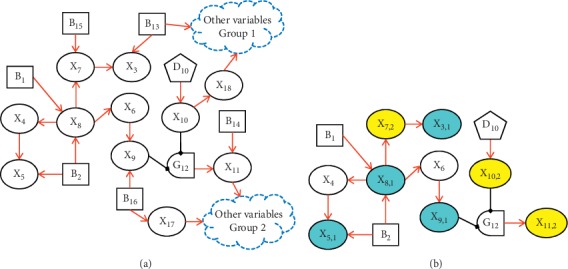
Example of DUCG diagnostic causality graph: (a) original causality graph and (b) simplified causality graph.

The DUCG graphically and compactly represents the events and causalities among events.  Figure 1 shows an example of DUCG. An *X*-type variable (oval-shaped node) represents an observable effect event, which can include medical information such as symptoms, signs, and examination findings. A *B*-type variable (square-shaped node) denotes a root cause event or a disease origin. A *G*-type (logic gate node) variable represents the combinational logic relations between its inputs and outputs (e.g., *G*_12_ in Figure 1(a) with its state expression specified in Table 1). A *D*-type variable (pentagon node) represents the default or unspecified cause of an *X*-type variable. A certain state *j* of variable *V*_*i*_ (*V* can be a *B*-, *X*-, *G*-, or *D*-type), referred to as *V*_*i*,*j*_, represents an event; state number *j* = 0 indicates a normal state and *j* ≠ 0 indicates an abnormal state.

The arc *V*_*i*,*j*_⟶*X*_*n*,*k*_ represents a virtual functional event *F*_*n*,*k*;*i*,*j*_ ≡ (*r*_*n*;*i*_/*r*_*n*_)*A*_*n*,*k*;*i*,*j*_ that describes the causality between a child event *X*_*n*,*k*_ and a parent event *V*_*i*,*j*_. *A*_*n*,*k*;*i*,*j*_ denotes the independent causality function that *V*_*i*,*j*_ causes *X*_*n*,*k*_. The intensity of causal relationship between *X*_*n*_ and *V*_*i*_ is defined as *r*_*n*;*i*_, and *r*_*n*;*i*_ ≥ 0, *r*_*n*_ ≡ ∑_*i*_*r*_*n*;*i*_. As a weighting factor of *A*_*n*;*i*_, the coefficient (*r*_*n*;*i*_/*r*_*n*_) balances these independent causality functions for all parent events on a same child event. For *A*/*F*/*a*-type variables, the variable subscripts are in the format of “child; parent,” and the variable identifiers in lower case letters denote the probability parameters of the variables in corresponding upper case letters, for example, *a*_*n*,*k*;*i*,*j*_=*Pr*{*A*_*n*,*k*;*i*,*j*_} and *b*_*i*,*j*_=*Pr*{*B*_*i*,*j*_}.

The causality representation mechanism of DUCG is a *weighted logic event expansion*, which is denoted below:

(1) $$\begin{array}{c} X_{n,k}=\underset{i}{\sum }F_{n,k;i}V_{i}=\underset{i}{\sum }\frac{r_{n;i}}{r_{n}}\underset{j}{\sum }A_{n,k;i,j}V_{i,j}. \end{array}$$

A child event *X*_*n*,*k*_ can be logically expanded into a weighted sum of independent causality functions from all its parent events *V*_*i*,*k*_. Thus, the multivalued causalities between child-parent events in each pair are independently represented, rather than using joint probability distribution as in the other models such as BN. The use of a compact causality representation accords the intuitive cognition of people to the real world and is more explicable. For DUCG, it is convenient to represent the causalities and parameters based on findings of medical research and clinical knowledge, or statistical learning from clinical sample data.

#### 2.1.2. DUCG-Based Diagnostic Reasoning Method

The diagnostic inference method includes three steps: (1) simplifying the original causality graph based on observed evidence; (2) weighted logic event expansion and logical reasoning for evidential events; and (3) probabilistic reasoning. If *H*_*k,j*_ represents a candidate hypothesis, i.e., a possible root cause (disease origin) of evidence *E*, then the state probability of *H*_*k,j*_, conditioned on *E* and denoted by *h*_*k*,*j*_^*s*^, is calculated as

(2) $$\begin{array}{c} h_{k,j}^{s}=\mathrm{Pr}\left\{H_{k,j}\left|E\right.\right\}=\frac{\mathrm{Pr}\left\{H_{k,j}E\right\}}{\mathrm{Pr}\left\{E\right\}}, \end{array}$$

where *E* is defined by *E*=∏_*n*_*X*_*n*,*k*_. Every evidence event *X*_*n*,*k*_ is independently expanded into logic expressions according to (1) by tracing their upstream causality chains back towards the root cause event(s), respectively. Such an inference process is called *chaining inference*. Meanwhile, some basic logic operations, including *OR*, *AND*, *NOT*, *XOR*, *absorption*, *exclusion* (events are mutually incompatible), and *complementation*, are performed. Thus, the candidate hypothesis space *S*_*H*_ = {*H*_*k*,*j*_} can be obtained. Finally, the posterior probabilities of the hypothesis event *H*_*k*,*j*_ can be calculated. In case of more than one candidate hypotheses in *S*_*H*_, the state probability of *H*_*k*,*j*_ is modified by the weight coefficient defined on the prior probability of evidence (referred to as *ζ*_*k*_, *ζ*_*k*_ ≡ *Pr*{*E*}) in the causality context of each hypothesis *H*_*k*,*j*_:

(3) $$\begin{array}{c} h_{k,j}^{s}=h_{k,j}^{s}\frac{\zeta_{k}}{\sum_{k}\zeta_{k}}. \end{array}$$

The probabilistic normalization of weighted logic event expanding expressions, i.e., ∑_*k*_*Pr*{*X*_*n*,*k*_}=∑_*k*_∑_*i*_(*r*_*n*;*i*_/*r*_*n*_)∑_*j*_*a*_*n*,*k*;*i*,*j*_*v*_*i*,*j*_=1, holds automatically because ∑_*i*_(*r*_*n*;*i*_/*r*_*n*_)=1, ∑_*k*_*a*_*n*,*k*;*i*,*j*_=1, and ∑_*j*_*v*_*i*,*j*_=1. By virtue of such an *automatic normalization* feature, the chaining inference can be proven as *self-reliant* such that *Pr*{*X*_*n*,*k*_} is calculated without the use of *Pr*{*X*_*n*,*k*′_}(*k*′ ≠ *k*), and the irrelevant parameters (e.g., *a*_*n*,*k*′;*i*,*j*_) can be absent without affecting the inference accuracy. Moreover, the variation of parameter values in the numerator of (2) corresponds to the variation in the parameter values in the denominator. It is thus the ratio of the parameter values in the event expanding expressions of *Pr*{*H*_*k*,*j*_*E*}, to that of *Pr*{*E*} that has an actual effect on the final result. Based on the aforementioned features, the exact inference can be used in case of incomplete and inaccurate parameters and clinical data.

The causality graph in Figure 1 can be used as a calculation case to clarify the details of the diagnostic inference algorithm. Let us suppose that the parameter matrices are given as follows (for the sake of simplicity, the weighting factors *r*_*n;i*_ are all set to 1). For a matrix element *a*_*n*,*k*;*i*,*j*_ (e.g., *a*_3,1;7,1_, *a*_5,1;2,2_, and *a*_6,2;8,1_), *k* and *j* correspond to the row number and column number of the matrix, respectively. The symbol “−” indicates unknown, unavailable, or irrelevant parameters:

(4) $$\begin{array}{c} b_{1}=\left(\begin{array}{c} -\\ 0.02 \end{array}\right),\\ b_{2}=\left(\begin{array}{c} -\\ 0.02\\ 0.04 \end{array}\right),\\ a_{3;7}=\left(\begin{array}{ccc} - & - & -\\ - & 0 & 0.5 \end{array}\right),\\ a_{4;8}=\left(\begin{array}{cc} - & -\\ - & 0.6 \end{array}\right),\\ a_{5;2}=\left(\begin{array}{ccc} - & \;- & -\\ - & 0.2 & 0 \end{array}\right),\\ a_{5;4}=\left(\begin{array}{cc} - & -\\ - & 0.8 \end{array}\right),\\ a_{6;8}=\left(\begin{array}{cc} - & -\\ - & 0.6\\ - & 0 \end{array}\right),\\ a_{7;8}=\left(\begin{array}{cc} - & -\\ - & 0\\ - & 0.9 \end{array}\right),\\ a_{8;1}=\left(\begin{array}{cc} - & -\\ - & 0.9 \end{array}\right),\\ a_{8;2}=\left(\begin{array}{ccc} - & \;- & -\\ - & 0.6 & 0 \end{array}\right),\\ a_{9;6}=\left(\begin{array}{ccc} - & \;- & -\\ - & 0.6 & 0 \end{array}\right),\\ a_{10;10\;D}=\left(\begin{array}{c} -\\ 0.2\\ 0.8 \end{array}\right),\\ a_{11;12}=\left(\begin{array}{cc} - & -\\ - & 0\\ - & 0.9 \end{array}\right). \end{array}$$

Besides the normal or unknown variables, suppose that the evidence for the DUCG example in Figure 1(a) is obtained as *E*=∏_*i*_*E*_*i*_′=*X*_3,1_*X*_5,1_*X*_7,2_*X*_8,1_*X*_9,1_*X*_10,2_*X*_11,2_. By applying the simplification rules of DUCG [28, 30], all the irrelevant, incorrect, and meaningless causalities and events can be eliminated from the graph based on the medical evidence. Thus, the simplified graph is obtained as shown in Figure 1(b). DUCG uses different colored nodes to distinguish the *X*-type variables' states: green indicates a normal state indexed by 0, sky blue and blue indicate state 1 and state 3, respectively, yellow and brown indicate state 2 and state 4, respectively, and colorless indicates an unknown state, implying an unconfirmed/unidentifiable pathological feature or an unexamined item.

Based on (1) and Figure 1(b), the expanding expressions of evidence *X*_3,1_, *X*_5,1_, and *X*_11,2_ in the causality context of *B*_2,1_, respectively, are shown below:

(5) $$\begin{array}{c} E_{1}^{\prime }=X_{3,1}=A_{3,1;7,2}A_{7,2;8,1}A_{8,1;2,1}B_{2,1},\\ E_{2}^{\prime }=X_{5,1}=\left(\frac{1}{2}\right)A_{5,1;2,1}B_{2,1}+\left(\frac{1}{2}\right)A_{5,1;4,1}A_{4,1;8,1}A_{8,1;2,1}B_{2,1},\\ E_{7}^{\prime }=X_{11,2}=A_{11,2;12,1}A_{10,2;10\;D}A_{9,1;6,1}A_{6,1;8,1}A_{8,1;2,1}B_{2,1}. \end{array}$$

Likewise, other abnormal evidence is expanded, and the expanding expression for *E* is obtained as

(6) $$\begin{array}{c} E=A_{3,1;7,2}A_{7,2;8,1}A_{11,2;12,1}A_{10,2;10\;D}A_{9,1;6,1}A_{6,1;8,1}A_{8,1;2,1}B_{2,1}\cdot \left(\left(\frac{1}{2}\right)A_{5,1;2,1}B_{2,1}+\left(\frac{1}{2}\right)A_{5,1;4,1}A_{4,1;8,1}A_{8,1;2,1}B_{2,1}\right)=\left(\frac{1}{2}\right)A_{3,1;7,2}A_{7,2;8,1}A_{11,2;12,1}A_{10,2;10\;D}A_{9,1;6,1}A_{6,1;8,1}A_{8,1;2,1}A_{5,1;2,1}B_{2,1}+\left(\frac{1}{2}\right)A_{3,1;7,2}A_{7,2;8,1}A_{11,2;12,1}A_{10,2;10\;D}A_{9,1;6,1}A_{6,1;8,1}A_{5,1;4,1}A_{4,1;8,1}A_{8,1;2,1}B_{2,1}. \end{array}$$

Similarly, the expanding expression of *E* in the causality context of *B*_1,1_ is

(7) $$\begin{array}{c} E=A_{3,1;7,2}A_{7,2;8,1}A_{11,2;12,1}A_{10,2;10\;D}A_{9,1;6,1}A_{6,1;8,1}A_{5,1;4,1}A_{4,1;8,1}A_{8,1;1,1}B_{1,1}. \end{array}$$

Finally, hypothesis space *S*_*H*_ under evidence *E* is obtained as follows: *S*_*H*_={*H*_1,1_, *H*_2,1_}, where *H*_1,1_ ≡ *B*_1,1_ and *H*_2,1_ ≡ *B*_2,1_. Therefore, the two hypotheses are determined as the probable causes of the observed evidence, as both can independently interpret all the symptoms. The probabilistic calculation results are *h*_1,1_^*s*^=0.679 and *h*_2,1_^*s*^=0.321. The inference results can be explained using the graphical causality semantics as shown in Figure 1(b). Based on these semantics, users can not only deduce the results but also understand their logic.

Based on the self-reliant chaining inference, the missing parameters, such as *a*_3,1;7,2_, *a*_7,2;8,1_, *a*_11,2;12,1_, *a*_10,2;10*D*_, *a*_9,1;6,1_, and *a*_6,1;8,1_, do not have an impact on the reasoning result. Moreover, the most probable hypotheses could be typically determined uniquely, just within the logical reasoning process (before the probabilistic reasoning is performed). Thus the causality structure that accurately represents the disease pathogenesis is what really matters. It is evident that DUCG does not impose stringent requirements for completeness of the parameters and clinical examination data. Thus, it can be used to facilitate modeling through medical knowledge and experience.

### 2.2. Construction of Differential Diagnosis Model for BPPV

#### 2.2.1. BPPV Characteristics

*(1) Pathophysiology of BPPV*. The vestibular receptor consists of two otolith organs (the utricle and saccule), which oversee linear acceleration and gravity, as well as three semicircular canals (SCCs) that sense angular acceleration. The SCCs include anterior SCCs (ASCCs), posterior SCCs (PSCCs), and lateral SCCs (LSCCs). The afferent nerves from otolith organs and SCCs project symmetrical activities to the central vestibular system to maintain the balance and spatial orientation. BPPV results from misplaced calcium carbonate crystals (otoconia) that are detached from utricle macula and collected within the SCCs due to trauma, infection, or even aging. These crystals either remain free-floating in the SCCs or become attached to the cupula. Normally, as the motion sensor of SCCs that are filled with endolymph, cupula is a gelatinous mass with the same density as endolymph. Since the calcium particles are denser than the endolymph and cupula, the SCCs become pathologically sensitive to linear acceleration and gravity; the afferent nerves from SCCs thus fire asymmetrically, leading to vertigo and nystagmus. When moving into the SCC, the calcium carbonate crystal debris may cause endolymph movement, which consequently stimulates the cupula of the affected canal during head movement or while stationary (this is called canalithiasis). Similarly, if adherent to the cupula, the particles may also activate it (this is called cupulolithiasis) [32].

*(2) Clinical Characteristics of BPPV*. BPPV is characterized by brief attacks of vertigo after lying down in bed, looking up or bending down, among others. The vertigo attacks in most cases last less than 1∼2 minutes and mostly occur at night or upon awakening [1]. The diagnosis rests on the observation of characteristic nystagmus accompanying symptoms of vertigo when a patient's head is moved into a specific orientation with respect to gravity. This is due to shred calcium crystals from macula, which may cause age-related changes in the protein and gelatinous matrix of the otolithic membrane. Although most of BPPV cases are idiopathic, a significant proportion can be associated with preceding traumatic events including head trauma, dental treatment, and ear surgery. Other conditions, such as viral vestibular neuritis, otitis media, Ménière's disease, idiopathic sudden sensorineural hearing loss, posterior circulation ischemia, and migraine, can also trigger cases of BPPV.

BPPV may affect each of the three SCCs. Alternatively, it can affect more than one canal simultaneously, resulting in varying nystagmus patterns [33]. Notably, due to its gravity-dependent position, the most commonly affected semicircular canal is the posterior canal. The positional and positioning tests (e.g., Dix–Hallpike diagnostic maneuver and supine roll test) can provide an accurate diagnosis for this condition. Furthermore, the features of nystagmus, including latency, direction, duration, reversal, and fatigability, are significant during diagnosis.

*(3) Subtype Differentiation of BPPV*. Based on the epidemiology, causes of disease, pathophysiology, anatomy pathology, clinical features, and treatment outcomes, a BPPV disease may be classified into distinct categories along four dimensions: (1) depending on the localization of the particles and the involved semicircular canals, there are four subtypes (PSCC, ASCC, LSCC, and multiple-SCC (MSCC)); (2) BPPV is subdivided into idiopathic BPPV and secondary BPPV, as it may occur as a primary disease or secondary to other otology disorders or systemic conditions [4]; (3) BPPV can be divided into canalithiasis and cupulolithiasis, depending on the nystagmus provoked characteristics from the positioning test based on the pathophysiology mechanism [32]; and (4) based on the clinical features and treatment outcomes, BPPV falls into two categories: typical and atypical. The atypical BPPV can be further identified as subjective BPPV, persistent BPPV, and recurrent BPPV. Subjective BPPV refers to the cases associated with a positive history of BPPV and Dix–Hallpike; or supine roll tests which are positive for vertigo, but negative for nystagmus [34]. A persistent BPPV refers to cases lasting for more than two weeks, while a recurrent BPPV indicates recurrence of BPPV in the same canal after a symptom-free interval of at least two weeks from a previously successful treatment [35].

#### 2.2.2. Constructed Model for Differential Diagnosis of BPPV

**Figure 2:**
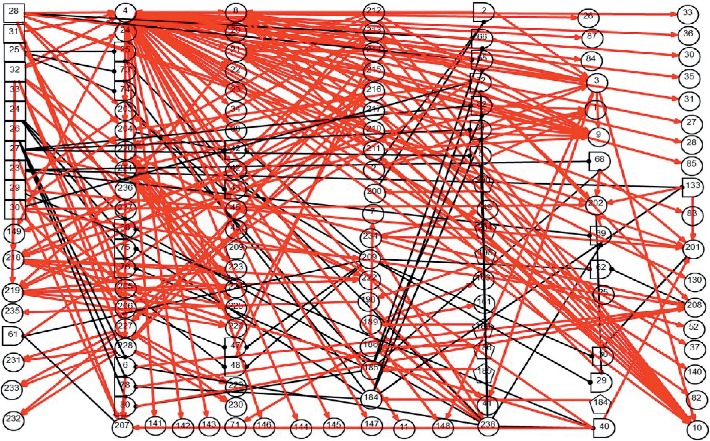
The DUCG-based differential diagnostic causality graph for BPPV.

As a causality representation to the aforementioned pathogenesis and pathophysiology of BPPV, a new DUCG-based causality graph was constructed based on the vertigo model presented in [30]. The BPPV differential diagnosis model is shown in Figure 2; it includes 125 variables (*X*-type: 85, *D*-type: 8, *G*-type: 21, and *B*-type: 11) and 286 arcs.

**Figure 3:**
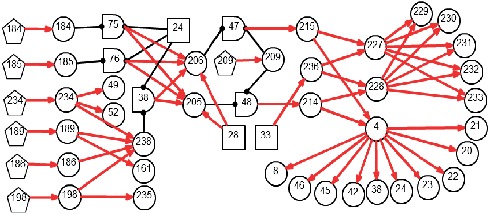
Sub-DUCG for LSCC-idiopathic-recurrent BPPV.

**Figure 4:**
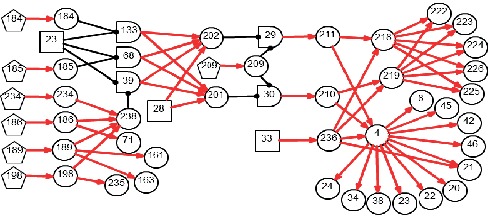
Sub-DUCG for PSCC-idiopathic-recurrent BPPV.

**Figure 5:**
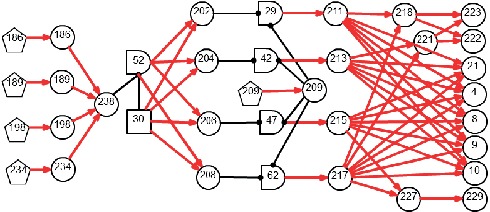
The sub-DUCG for cupulolithiasis BPPV.

*(1) Modularized Modeling Scheme*. A modularized modeling scheme is applied in the construction of the causality graph shown in Figure 2. All BPPV subtypes are handled as individual sub-DUCGs. For example, the three sub-DUCGs representing the idiopathic, PSCC, LSCC, and cupulolithiasis BPPV are demonstrated in Figures 3–5, respectively. When the sub-DUCGs are merged, solutions are proposed for addressing the ambiguous, contradictory, and incomplete information to ensure global coherence [30]. Such a modularized modeling scheme reduces the difficulty in model construction using a divide-and-conquer approach.

*(2) Definition of Variables*. Based on the characteristics of BPPV and the international diagnostic criteria [36], the medical information of patients, including symptoms, signs, findings of examinations, medical histories, etiology and pathogenesis, pathophysiology, and socio-psychological and environmental factors is incorporated into the model.  Table 2 lists some of the variables of the model shown in Figure 2.

*(3) Representation of Causal Relationships*. In this representation, arcs are established to represent and quantify causal correlations among related symptoms and disease origins; complex causalities are denoted by a combination of causal functions via logic gates. All causalities and parameters in Figure 2 are determined by otoneurologists, based on their knowledge, experience, epidemiology statistics, and research achievements. As stated before, both the incompleteness of knowledge and imprecision of parameters have little impact on the reasoning accuracy; thus, DUCG is more flexible for the model construction.

### 2.3. The Algorithm of Differential Diagnostic Reasoning

Differential diagnosis aims at narrowing down the diseases to the most probable one from a list of candidate diseases that show similar symptoms. The differential diagnostic reasoning algorithm is presented as Algorithm 1, which implements a hypothesis-driven process. The posterior probability of the hypothesis event *H*_*k*,*j*_ is used to quantify *H*_*k*,*j*_'s ability to interpret the medical evidence, *E*. Notably, a rare disease does not necessarily respond to a weak causality. If a rare disease (with a low prior probability) better explains a patient's symptoms and medical evidence, then the strength of the causality functions can be high; thus, the disease will get a higher ranked probability as a hypothesis event.

#### 2.3.1. New Causality Simplification Mechanisms

Based on the collected medical evidence *E* for a patient, the process of causality simplifications is performed to the BPPV causality graph. Causality simplification aims to eliminate the irrelevant, meaningless, and impossible events and relations from a causality graph. Therefore, the model scale and complexity are both significantly decreased, and the disease hypothesis space converges. The root cause event can even be preliminarily determined based on the simplified causality graph.

As a complement to the original simplification rules of DUCG [28, 30], several new causality simplification mechanisms, including a pruning strategy and causality tracking process, are presented.

**Definition 1 (Pruning Strategy).**

The meaningless disease hypothesis and its corresponding causalities are identified and pruned from the causality graph using a scheme of causality tracking for all the abnormal evidences.

**Definition 2 (Causality Tracking Process).**

The causality tracking process starts from an observed event and traces its upstream causality chains and ancestors back towards the *B*-type event(s). For any *B*_*i*_, only if all the abnormal evidence is reachable to it via a causality tracking process, can it be identified as a candidate hypothesis; otherwise, *B*_*i*_ should be eliminated from the hypothesis space, and its corresponding causalities should be pruned from the graph.

#### 2.3.2. Weighted Logic Inference Rules of Differential Diagnosis for BPPV

Based on the simplified causality graph and medical evidence, the process of logical inference is performed to obtain the hypothesis space according to (1)∼(3). Our earlier study showed that by applying event expanding and logical operations, the logical inference can effectively decrease the complexity of probabilistic reasoning [28]. However, in response to BPPV differential diagnosis, the weighted logic inference algorithm requires major modifications.

Some BPPV subtypes can be present in a patient simultaneously. For instance, a diagnosis can be termed as an ASCC-idiopathic-canalithiasis BPPV. Such a case is known as *concurrency of subtypes*. Meanwhile, the subtypes belonging to the same category are mutually exclusive when a diagnostic conclusion is drawn. Some examples of this are PSCC-BPPV (*B*_23,1_), LSCC-BPPV (*B*_24,1_), ASCC-BPPV (*B*_25,1_), and MSCC-BPPV (*B*_26,1_). Therefore, to deal with these cases, new weighted logic inference rules for exclusion and concurrency, among others, are developed as shown below.

**Rule 1 .**

Suppose that the basic events (namely, root cause events) in a BPPV differential diagnosis model are represented in terms of category sets as *C*_1_ = {*B*_23,1_, *B*_24,1_, *B*_25,1_, *B*_26,1_}, *C*_2_ = {*B*_27,1_, *B*_28,1_}, *C*_3_ = {*B*_29,1_, *B*_30,1_}, and *C*_4_ = {*B*_31,1_, *B*_32,1_, *B*_33,1_}. State 1 of a *B*-type event indicates that the root cause event has occurred; on the contrary, state 0 of a *B*-type event indicates that the root cause event has not occurred. The mutual exclusion rules for basic events in BPPV differential diagnosis can thus be defined as *B*_*i*,1_*B*_*i*′,1_=0, where *B*_*i*,1_, *B*_*i*′,1_ ∈ *C*_*m*_(*i* ≠ *i*′, *m* ∈ {1,2,3}).

**Proof**

From the definition, the category sets *C*_1_, *C*_2_, and *C*_3_ indicate different classification perspectives of BPPV. Therefore, all basic events within the same category set are exclusive and exhaustive. Specifically, there is *B*_23,1_*B*_24,1_ = 0, *B*_23,1_*B*_25,1_ = 0, *B*_23,1_*B*_26,1_ = 0, *B*_24,1_*B*_25,1_ = 0, *B*_24,1_*B*_26,1_ = 0, *B*_25,1_*B*_26,1_ = 0, *B*_27,1_*B*_28,1_ = 0, and *B*_29,1_*B*_30,1_ = 0.

**Rule 2 .**

*B*_*i*,1_ *B*_*i*′,1_ ≠ 0 for *B*_*i*_, *B*_*i*′_ ∈ *C*_4_ (*i* ≠ *i*′) and *B*_*i*,1_*B*_*i*′,1_ ≠ 0 for *B*_*i*,1_ ∈ *C*_*m*_, *B*_*i*′,1_ ∈ *C*_*n*_(*i* ≠ *i*′, *m* ≠ *n*).

**Proof**

The events in *C*_4_ describe the respective different types of atypical BPPV, in an overlapping manner. Namely, all the basic events in {*B*_31,1_, *B*_32,1_, *B*_33,1_} are likely to be concurrent. This sets the basis for Rule 2.

**Definition 3 (Maximizing Concurrent Basic Event Set Strategy).**

To obtain an ultimate disease hypothesis *H*_*k*,*j*_, if the events that constitute *H*_*k*,*j*_ are concurrent and consistent with each other, the maximum number of basic events within the hypothesis space *S*_*H*_ should be incorporated.

It is essential to explore every possible outcome before ruling out any basic event. For example, as *S*_*H*_ = {*B*_24,1_, *B*_28,1_, *B*_30,1_} is obtained for a case, we should get only the correct hypothesis *H*_1,1_ = *B*_24,1_*B*_28,1_*B*_30,1_ ≠ 0, in which the three events together interpret the observed symptoms, rather than *B*_24,1_*B*_28,1_, *B*_28,1_*B*_30,1_, or so on.

**Rule 3 .**

Given *V* ∈ {*B*,  *X*,  *G*,  *D*}, *j* ≠ *j*′, and integer *y* ≥ 2, then, (*V*_*ij*_)^*y*^=*V*_*ij*_ and *V*_*ij*_*V*_*ij*_=0.

**Rule 4 .**

Given integer *y* ≥ 2, *k* ≠ *k*′, and *j* ≠ *j*′, then, (*F*_*nk*;*ij*_)^*y*^=(*r*_*n*;*i*_/*r*_*n*_)^*y*^*A*_*nk*;*ij*_, *F*_*nk;ij*_*F*_*nk′;ij*_ = 0, *F*_*nk*;*ij*_*F*_*nk*;*ij′*_ = 0, and *F*_*nk*;*ij*_*F*_*nk′*;*ij′*_ = 0; if *B*_*i*,1_*B*_*i*′,1_=0 is given, *B*_*i*,1_, *B*_*i*′,1_ ∈ *C*_*m*_, *i* ≠ *i*′, *m* ∈ {1,2,3}, then it follows that, *F*_*nk*;*i*′,1_*B*_*i*,1_ = 0, *F*_*nk*;*i*,1_*B*_*i*′,1_ = 0, and *F*_*nk*;*i*,1_*F*_*nk*;*i*′,1_ = 0.

**Proof**

The latter part of Rule 4 is based on Rule 1, and the other part has been proven in [28]. From Rule 1, *B*_*i*,1_*B*_*i*′,1_=0 which implies that the basic events *B*_*i*,1_ and *B*_*i*′,1_ cannot be concurrent and consequently brings about the mutual exclusions among the function events related to *B*_*i*,1_ and *B*_*i*′,1_. Therefore, *A*_*nk*;*i*,1_*A*_*nk*;*i*′,1_ = 0 and *F*_*nk*;*i*,1_*F*_*nk*;*i*′,1_ = 0, as *A*_*nk*;*i*,1_ cannot be present in combination with *A*_*nk*;*i*',1_; similarly then, *F*_*nk*;*i*′,1_*B*_*i*,1_ = 0 and *F*_*nk*;*i*,1_*B*_*i*′,1_ = 0. Notably, *F*_*nk*;*i*,1_*F*_*nk*;*i*′,0_ ≠ 0 is true because *A*_*nk*;*i*,1_ and *A*_*nk*;*i*′,0_ are independent of each other (since they are given independently).

**Rule 5 .**

∑_*m*=1_^*M*^∏_*i*∈*S*_*m*__*F*_*nk*;*ij*_*i*__*V*_*ij*_*i*__=(∑_*m*=1_^*M*^∏_*i*∈*S*_*m*__(*r*_*n*;*i*_/*r*_*n*_))∏_*i*∈*S*_1__*A*_*nk*;*ij*_*i*__*V*_*ij*_*i*__, where *S*_*m*_ denotes the set of variable number in a product item, *m *∈ {1,2,…, *M*} and *S*_1_⊆*S*_2_⊆⋯⊆*S*_*M*_.

**Rule 6 .**

Suppose that no mutually exclusive basic events coexist in the candidate hypothesis space, then, *F*_*nk*;*ij*_*V*_*ij*_(∑_*i*′_*F*_*nk*;*i*′*j*_*i*′__*V*_*i*′*j*_*i*′__)=*F*_*nk*;*ij*_*V*_*ij*_, if *j* = *j*_*i*_ is given.

**Proof**

To complement the ordinary conditions of Rule 14 discussed in [28], consider a situation related to mutually exclusive basic events, *V*_*ij*_ = *B*_*i*,1_ and *V*_*ij*′_ = *B*_*i*′,1_, *B*_*i*,1_, *B*_*i*′,1_ ∈ *C*_*m*_(*i* ≠ *i*′, *m* ∈ {1,2,3}) as proposed in Rule 1. The proposition of Rule 6 can thus be represented as

(8) $$\begin{array}{c} F_{nk;ij}V_{ij}\left(\underset{i^{\prime }}{\sum }F_{nk;i^{\prime }j_{i^{\prime }}}V_{i^{\prime }j_{i^{\prime }}}\right)=F_{nk;ij}V_{ij}\cdot F_{nk;ij}V_{ij}+F_{nk;ij}V_{ij}\cdot \underset{i^{\prime }\neq i}{\sum }F_{nk;i^{\prime }j_{i^{\prime }}}V_{i^{\prime }j_{i^{\prime }}}\\ ={\left(r_{n;i}/r_{n}\right)}^{2}A_{nk;ij}V_{ij}\\ \neq F_{nk;ij}V_{ij}. \end{array}$$

The inclusion of mutually exclusive events leads to the aforementioned outcomes (i.e., the inconsistence between Rule 1 and Rule 6), and the autonormalization feature no longer holds. Therefore, a solution for BPPV subtype differentiation known as a scheme of dummy basic variable (DBV) is proposed.

#### 2.3.3. Scheme of Dummy Basic Variable

**Definition 4 (DBV).**

If mutually exclusive candidate hypotheses coexist in the candidate hypothesis space, i.e., *B*_*i*,1_*B*_*i*′,1_=0, *B*_*i*,1_, *B*_*i*′,1_ ∈ *C*_*m*_(*i* ≠ *i*′, *m* ∈ {1,2,3}), *B*_*i*,1_ and *B*_*i*′,1_ are converted into different states of a DBV; each state of the DBV elicits an individual pathogenesis causality graph according to the hypothesis related to that state; the specific causalities of different diseases can be distinguished by the *A*-parameter values of DBV, which can either be zero or not. The exclusive rules among basic events are thus transformed into an inherent exclusive law among the states of an event.

**Figure 6:**
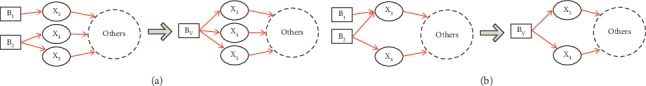
Example of DBV. (a) Example 1. (b) Example 2.

The problem of disobeying the autonormalization feature can thus be addressed. This method is illustrated via an example in Figure 6(a). Suppose that *B*_1,1_ and *B*_2,1_ are mutually exclusive events. They are then combined into a DBV-*B*_*v*_ during the reasoning process, corresponding to *B*_*v*,1_ and *B*_*v*,2_, respectively. From the definition of DBV, *a*_3,1;*v*,1_ ≡ *a*_3,1;1,1_, *a*_4,1;*v*,2_ ≡ *a*_4,1;2,1_, *a*_5,1;*v*,2_ ≡ *a*_5,1;2,1_, and *r*_*n*;*v*_ ≡ *r*_*n*;*i*_, where *i* ∈ {1,2} and *n* ∈ {3,4,5}.  Figure 6(b) shows an example where an event (e.g., *X*_3_) is connected to more than one basic event. In this case, *r*_*n*;*v*_ is defined as *r*_*n*;*v*_ ≡ ∑_*i*_*r*_*n*;*i*_, if *F*_*n*;*i*_ ≠ 0. Thus, *r*_3;*v*_ = *r*_3;1_ + *r*_3;2_ for the case in Figure 6(b) which points out to a combination of basic events.

In the context of differential diagnosis, and upon the completion of causality simplifications, the obtained candidate hypotheses of *C*_1_ = {*B*_23,1_, *B*_24,1_, *B*_25,1_, *B*_26,1_} (there are more than one basic event in the set), should be combined into a DBV-*B*_*m*_, with *B*_*m*,*k*_ denoting *B*_*i*,1_, where *k* = 1, 2, 3, 4 and *i* = 23, 24, 25, 26; Pr{*B*_*m*,*k*_} refers to the prior probability of *B*_*i*,1_, and *Pr*{*B*_*m*,0_}=1 − ∑_*k*≠0_*Pr*{*B*_*m*,*k*_}.

**Definition 5 (Ranked Independent Probability).**

The ranked independent probability represents a new metric proposed for quantifying the degree of confidence in whether a BPPV subtype (e.g., *B*_*i*,1_ as a part of the hypothesis *H*_*k,j*_) can independently interpret all the medical evidence, in comparison with the other subtypes coexisting in *H*_*k,j*_. The ranked independent probability of *B*_*i*,1_ is calculated as follows: (1) get *h*_*i*,1_^*s*^ of *B*_*i*,1_ according to (2) and (3), in which *ζ*_*i*_ is based on the individual causality tracking graph of *B*_*i*,1_; and (2) for all the *B*_*i*,1_ within the set of *H*_*k,j*_, *h*_*i*,1_^*s*^ is ranked by *h*_*i*,1_^*s*^/∑_*i*_*h*_*i*,1_^*s*^.

### 2.4. Theoretical Analysis of the Reasoning Complexity

The inference algorithm for the proposed method reduces the reasoning complexity in two ways. First, the causality graph simplification and pruning strategy significantly decreases the model scale. The variables that are irrelevant or inconsistent with the medical evidence are excluded from the reasoning calculation. This significantly reduces the computational complexity. Moreover, the diagnostic reasoning conclusion can even be accurately drawn solely via the causality simplification. Second, prior to probabilistic reasoning, weighted logic inference is performed based on the simplified causality graph. Thus, the multiple connected causalities can be decomposed independently, leading the chaining inference to exhibit high efficiency. Furthermore, the proposed logical inference rules and logical operations can decrease the complexity and scale of logical event expressions.

The actual efficiency tests for the DUCG inference algorithm have been performed using online fault diagnosis data from several large-scale industrial systems (e.g., nuclear power plants), in which thousands of variables and causality arcs are involved [28, 31]. The diagnostic reasoning can typically be finished within 0.5–1 s (on a personal computer). Moreover, the inference efficiency has been verified from our past clinical diagnosis applications that involved dozens of diseases, such as jaundice and sellar region disease.

## 3. Experiments and Analyses

### 3.1. Clinical Validation of the BPPV Differential Diagnosis Method

#### 3.1.1. Differential Diagnosis for BPPV—Case 1

Suppose that the medical evidence of a patient was observed in part as {*X*_4,1_, *X*_21,2_, *X*_24,3_, *X*_184,4_, *X*_185,2_, *X*_209,4_, *X*_229,2_, *X*_230,2_, *X*_207,0_, *X*_208,0_, *X*_236,0_} in Figure 2 and other *X*-type variables are normal or unknown (incomplete medical information). The medical information in this experiment is from a 65-year-old female patient who suffered from brief episodes of vertigo when rolling over in bed for two weeks and was suspected to be a BPPV case according to the supine roll test.

**Figure 7:**
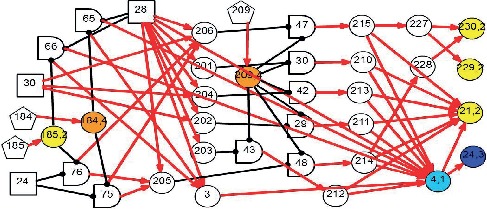
Diagnostic causality graph for Case 1.

**Figure 8:**
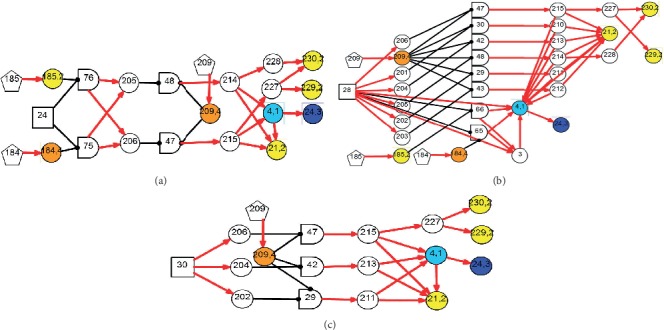
Individual causality tracking graphs of the hypotheses for Case 1. (a) LSCC-BPPV (B_24_). (b) Idiopathic BPPV (B_28_). (c) Cupulolithiasis BPPV (B_30_).

Based on the evidence, in this case, Figure 2 can be simplified into the graph shown in Figure 7, by applying the causality simplification rules and pruning strategy. Many meaningless relationships and events under current situation are eliminated accordingly, with *S*_*H*_ = {*B*_24,1_, *B*_28,1_, *B*_30,1_} being inferred as the possible root causes. The resulting hypothesis space, *S*_*H*_, is concise with all unlikely hypotheses being excluded from concern. Moreover, the individual causality tracking graphs for the three root cause events *B*_24_, *B*_28_, and *B*_30_ in Figure 7 are shown in Figure 8, whereby each subgraph independently covers all evidence. The graphs thus intuitively and faithfully represent the causalities underlying the pathogenesis of Case 1.

By applying the algorithm of differential diagnostic reasoning for Case 1, the state probability of *H*_1,1_ = *B*_24,1_*B*_28,1_*B*_30,1_ can be obtained as *h*_1,1_^*s*^=*Pr*{*H*_1,1_|*E*}=1. The obtained “ranked independent probabilities” of *B*_24,1_, *B*_28,1_, and *B*_30,1_ are listed in Table 3. The LSCC-idiopathic-cupulolithiasis BPPV is therefore determined as the diagnostic result of Case 1; this result is consistent with the clinical conclusions confirmed by otoneurologists.

#### 3.1.2. Differential Diagnosis for BPPV—Case 2

A 72-year-old female patient complained about paroxysmal positional vertigo for a month; the attacks of vertigo were brief, lasting for one minute; the vertigo frequently occurred while looking up or down. Thus, the medical evidence for this case is {*X*_4,1_, *X*_21,2_, *X*_24,3_, *X*_184,4_, *X*_185,2_, *X*_207,0_, *X*_208,0_, *X*_209,3_, *X*_222,3_, *X*_223,1_, *X*_224,1_, *X*_225,1_, *X*_226,1_, *X*_236,0_}; the other *X*-type variables are normal or unknown (incomplete medical information).

**Figure 9:**
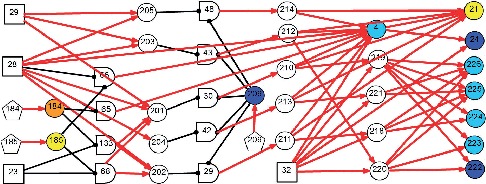
Diagnostic causality graph for Case 2.

By applying the algorithm of differential diagnostic reasoning, the hypothesis event *H*_1,1_ = *B*_23,1_*B*_28,1_*B*_29,1_*B*_32,1_ is uniquely determined as the origin of the disease, which indicates a PSCC-idiopathic-cupulolithiasis-persistent BPPV. The diagnostic causality graph is shown in Figure 9 which is based on the causality simplification of Figure 2. The resulting ranked independent probabilities of *B*_23,1_, *B*_28,1_, *B*_29,1_, and *B*_32,1_ are listed in Table 4. By explicitly representing the disease causes, symptoms, pathogenesis, and the latent correlations among medical information, Figure 9 offers intuitive insights into the underlying pathological mechanisms.

#### 3.1.3. Other Validated BPPV Subtype Cases

A verification experiment on some other clinical cases was performed to evaluate the effectiveness of the proposed method. The correctness was based on the otoneurologists' judgement on the diagnostic outcomes in contrast to the practical conclusions. In the clinical center for vertigo in Beijing Chaoyang Hospital, BPPV cases are common; however, there are multiple repetitions in the meaningful information for the BPPV patients on their medical histories and physical examinations regarding subtype differentiation. Based on this, 75 typical BPPV cases were selected from the thousands of cases containing medical records, interviews, and physical assessments in the verification experiments. The BPPV subtypes involved are outlined in Table 5. The selection principle of BPPV cases was based on the international diagnostic criteria for BPPV [36] and the typicality and representativeness of BPPV subtype diseases. Notably, since the nystagmus cannot be observed for a subjective BPPV case, it is difficult to classify the case either as canalithiasis BPPV or cupulolithiasis BPPV.

The diagnostic outcomes of the DUCG-based model agreed with the otoneurologists' conclusions with a correctness index of 100%. Based on the available published literature, no other research has reported a model-based automatic subtype differentiation of BPPV in the past.

### 3.2. Comparative Analysis of DUCG and BN

The main difference between DUCG and BN in terms of the theoretical framework is that the model structure and parameters of DUCG are decoupled. By virtue of such a feature, the independent causality representation by the weighted causality function event *A*_*n,k;i,j*_ in DUCG makes it possible to simplify the original causality graph based on the collected medical evidence. The irrelevant and meaningless events and relations are thus eliminated from the model. Taking the simplified causality graph as the basis of diagnostic reasoning reduces the computational scale and complexity to a large extent. In contrast, BN cannot be easily simplified owing to the structural coupling feature among the variables: the causalities among the child-parent events in BN are typically represented through a joint probability distribution (e.g., a conditional probabilistic table, CPT); in case any variables and relations are removed from a BN, the remaining parameters in the CPTs may need major adjustments; moreover, the number of parameters specified in a CPT for a BN is exponential to the number of the parent variables and states involved, while the parameters needed in the DUCG are less than the parameters in a BN.

Furthermore, by the independence representation and automatic normalization feature for the weighted logic event expansion, the “self-reliant” chaining inference and logic operations can be performed in DUCG. The parameters and information that are eliminated during the causality simplification and those that are not involved in the chaining inference can all be missing (or some parameters which are not of concern are thus not provided) during this reasoning process and do not affect the diagnostic accuracy. The diagnostic inference method under incomplete information indicates that some unnecessary clinical tests and inappropriate medications can be omitted, thereby decreasing the patients' medical costs. For a BN, the reasoning accuracy depends on the completeness of the parameters in the CPTs.

### 3.3. Application Analysis

The proposed method can be developed as an automatic diagnostic system for clinicians. Based on the compact, independent, and graphical causality representation using DUCG, the model construction and diagnosis application are both easy to implement. For a patient, the clinical data through can be obtained through inquiry, physical examination, and auxiliary examination, among others. Next, the clinical data, including symptoms, signs, examination, and test data, are input into the diagnostic reasoning interface of the system, and the diagnosis results can be obtained through reasoning calculation. The clinical application of this method is thus very convenient. Through incorporation into the automatic medical devices, this method could improve the efficiency of BPPV diagnosis and therapy, especially for general practitioners in primary health care institutions. In addition, the system can also be used in mobile and Internet terminals as a secondary tool for telemedicine; finally, it can serve as a teaching tool for doctor training in related disciplines.

## 4. Summary

This study proposes a DUCG method for differential diagnosis of BPPV. To distinguish among various causes of vertiginous disorders with similar symptoms, the proposed method involves a graphical and compact representation, in addition to logical reasoning algorithms for pathological mechanisms and related uncertain causalities. New algorithms of differential diagnostic reasoning are proposed by integrating a *pruning strategy* in the causality simplification process, a *maximizing concurrent basic event set strategy* in the formulation of hypothesis space, and the weighted logic inference rules for subtype differentiation of BPPV. Furthermore, a scheme of *dummy basic variable* is introduced to settle the problems related to mutually exclusive root cause events. The model manifests higher correctness, favorable robustness to incomplete evidence, and satisfactory discriminatory power for BPPV subtypes. Furthermore, using the diagnostic reasoning method and graphical representation mechanism not only provides a diagnostic result, but also explains the rationale for suggesting the diseases, which justifies the probability results.

In light of these encouraging preliminary results, more clinical studies will be performed. Furthermore, a new method of probabilistic prediction for the vestibular dysfunction-induced fall risk will be the subject of our future research endeavors.

## Figures and Tables

**Algorithm 1:**
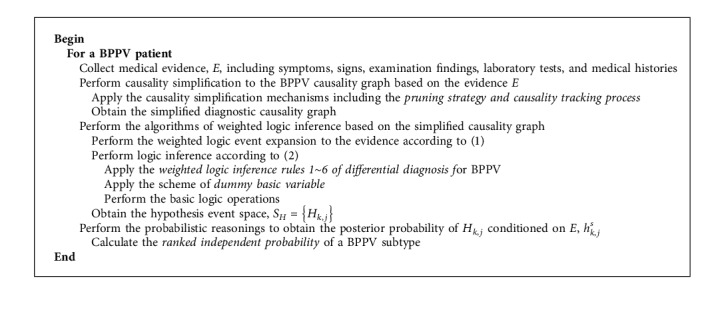
Differential diagnostic reasoning method for BPPV.

**Table 1:** Logic gate specification of *G*_12_.

Logic gate	State	State expression
*G*_12_	0	Remnant state
	1	*X*_9,1_ *X*_10,2_ + *X*_10,1_

**Table 2:** Definition of variables for differential diagnosis of BPPV.

Variables	Descriptions
*X*_1–3,201–206, 210–217_	Pathophysiology of BPPV: the lesion of the otolithic membrane of utricle macula, the degenerated otolith broken off from utricle macula, misplaced calcium carbonate crystals debris, calcium free-floating particles entering the SCCs, calcium particles adherent to the cupula of SCC, the endolymph movement with free-floating particles that pathologically stimulates the ampulla of canal, and the sensitivity to linear acceleration and gravity induced by the abnormal deflection of cupula
*X*_4,8–11_	The description of vertigo: an illusion of movement, the sensation that objects in the environment is moving when the eyes are open, and the sensation that a patient feels as if he or she is moving when the eyes are closed
*X*_11,42,45,46,82_	The main accompanying symptoms: spontaneous nystagmus and autonomic nerve symptoms
*X*_20-25_	The detailed description of the features of vertigo attacks: attack characteristics, the onset and duration of an attack, causes of disease, frequency, and severity
*X*_26–28,209_	Changes in head position relative to gravity: rotation of the head relative to the body while in an upright position
*X*_83–87,130_	The feature of spontaneous nystagmus and the Romberg test for vestibular function
*X*_52,49,161,163,238_	Other accompanying symptoms: cochlear symptoms, hearing loss, tinnitus, the symptoms of central nervous system, and the manifestations of the primary and underlying disorders
*X*_184,185_	Patient's gender and age
*X*_186,189,198,234_	The typical and characteristic medical history: a migraine, hypertension, head trauma, and inner ear pathology
*X*_207–208_	More than one semicircular canal is affected simultaneously (obtained statistically during the pretreatment process of medical data)
*X*_218–221,227,228_	Positioning test vertigo and nystagmus when the head is moving or rotating
*X*_222–226_	Dix–Hallpike test: the feature of evoked nystagmus (latency, duration, direction, amplitude, frequency, fatigability, and reversibility)
*X*_229–233_	Supine roll test: the feature of evoked nystagmus (latency, duration, direction, amplitude, frequency, fatigability, and reversibility)
*X*_236_	The outcomes of the maneuver treatment
*B*_23–33_	PSCC-BPPV (*B*_23_); LSCC-BPPV (*B*_24_); ASCC-BPPV (*B*_25_); MSCC-BPPV (*B*_26_); secondary BPPV (*B*_27_); idiopathic BPPV (*B*_28_); canalithiasis BPPV (*B*_29_); cupulolithiasis BPPV (*B*_30_); subjective BPPV (*B*_31_); persistent BPPV (*B*_32_); and recurrent BPPV (*B*_33_)

**Table 3:** Diagnostic reasoning results of Case 1.

BPPV subtype	Description	Ranked independent probability
*B*_24,1_	LSCC-BPPV	0.0388
*B*_28,1_	Idiopathic BPPV	0.9542
*B*_30,1_	Cupulolithiasis BPPV	0.007
*H*_1,1_	*B*_24,1_ *B*_28,1_ *B*_30,1_	1

**Table 4:** Diagnostic reasoning results of Case 2.

BPPV subtype	Description	Ranked independent probability
*B*_23,1_	PSCC-BPPV	0.1435
*B*_28,1_	Idiopathic BPPV	0.7653
*B*_29,1_	Canalithiasis BPPV	0.0091
*B*_32,1_	Persistent BPPV	0.0821
*H*_1,1_	*B*_23,1_ *B*_28,1_ *B*_29,1_ *B*_32,1_	1

**Table 5:** BPPV subtypes involved in the validated cases.

BPPV subtype	No. of cases
PSCC	53
ASCC	1
LSCC	16
MSCC	5
Idiopathic	55
Secondary	16
Canalithiasis	61
Cupulolithiasis	8
Subjective	4
Persistent	4
Recurrent	8
Typical	59

## Data Availability

The data used to support the findings of this study are selected from the clinical cases of Department of Otorhinolaryngology Head and Neck Surgery, Beijing Chaoyang hospital. Requests for access to these data should be made to Yanjun Wang (docwyjn@163.com, Beijing Chaoyang hospital).
